# The Tokyo Oldest Old Survey on Total Health (TOOTH): A longitudinal cohort study of multidimensional components of health and well-being

**DOI:** 10.1186/1471-2318-10-35

**Published:** 2010-06-09

**Authors:** Yasumichi Arai, Toshimitsu Iinuma, Michiyo Takayama, Midori Takayama, Yukiko Abe, Ryoko Fukuda, Jyuko Ando, Kikuko Ohta, Hiroo Hanabusa, Keiko Asakura, Yuji Nishiwaki, Yasuyuki Gondo, Hiroko akiyama, Kazuo Komiyama, Nobuhito Gionhaku, Nobuyoshi Hirose

**Affiliations:** 1 Division of Geriatric Medicine, Department of Internal Medicine, Keio University School of Medicine, 35 Shinanomachi, Shinjuku-ku, Tokyo 160-8582, Japan; 2 Department of Preventive Medicine and Public health, Keio University School of Medicine, 35 Shinanomachi, Shinjuku-ku, Tokyo 160-8582, Japan; 3 Faculty of Science and Technology, Keio University, 4-1-1 Hiyoshi, Kohoku-ku, Yokohama-shi, Kanagawa 223-8521, Japan; 4 Faculty of Environment and Information Studies, Keio University, 5322 Endo, Fujisawa-shi, Kanagawa 252-8520, Japan; 5 Faculty of Letters, 2-15-45 Mita, Minato-ku, Tokyo 108-8345, Japan; 6 Faculty of Nursing and Medical Care, Keio University, 5322 Endo, Fujisawa-shi, Kanagawa 252-8520, Japan; 7 Department of Complete Denture Prosthodontics, Nihon University School of Dentistry, 1-8-13 Kanda-Surugadai, Chiyoda-ku, Tokyo 101-8310, Japan; 8 Department of Pathology, Nihon University School of Dentistry, 1-8-13 Kanda-Surugadai, Chiyoda-ku, Tokyo 101-8310, Japan; 9 Osaka University Graduate School of Human Sciences, 1-2 Yamadaoka, Suita-shi, Osaka 565-0871, Japan; 10 The University of Tokyo, Institute of Gerontology, 7-3-1 Hongo, Bunkyo-ku, Tokyo 113-0033, Japan; 11 Hiro Clinic Shinjuku, 3-3-11 Nishi-Shinjuku, Shinjuku-ku, Tokyo 160-0023, Japan

## Abstract

**Background:**

With the rapid worldwide increase in the oldest old population, considerable concern has arisen about the social and economic burden of diseases and disability in this age group. Understanding of multidimensional structure of health and its life-course trajectory is an essential prerequisite for effective health care delivery. Therefore, we organized an interdisciplinary research team consisting of geriatricians, dentists, psychologists, sociologists, and epidemiologists to conduct a longitudinal observational study.

**Methods/Design:**

For the Tokyo Oldest Old Survey on Total Health (TOOTH) study, a random sample of inhabitants of the city of Tokyo, aged 85 years or older, was drawn from the basic city registry. The baseline comprehensive assessment consists of an in-home interview, a self-administered questionnaire, and a medical/dental examination. To perform a wide variety of biomedical measurements, including carotid ultrasonography and a detailed dental examination, participants were invited to our study center at Keio University Hospital. For those who were not able to visit the study center, we provided the option of a home-based examination, in which participants were simultaneously visited by a geriatrician and a dentist. Of 2875 eligible individuals, a total of 1152 people were recruited, of which 542 completed both the in-home interview and the medical/dental examination, with 442 completed the in-home interview only, and another 168 completed self or proxy-administered data collection only. Carotid ultrasonography was completed in 458 subjects, which was 99.6% of the clinic visitors (n = 460). Masticatory assessment using a colour-changeable chewing gum was completed in 421 subjects, a 91.5% of the clinic visitors.

**Discussion:**

Our results demonstrated the feasibility of a new comprehensive study that incorporated non-invasive measurements of subclinical diseases and a detailed dental examination aiming at community-dwelling individuals aged 85 years or older. The bimodal recruitment strategy is critically important to capture a broad range of health profiles among the oldest old. Results form the TOOTH study will help develop new models of health promotion, which are expected to contribute to an improvement in lifelong health and well-being.

**Trial Registration:**

This study has been registered in the UMIN-Clinical Trial Registry (CTR), ID: UMIN000001842.

## Background

One of the most striking features of modern society is the steady increase in life expectancy, accompanied by the rapid growth of the oldest old population, defined as those 85 years or older [1,2]. In Japan, where female life expectancy at birth reached 86 years in 2007, the number of the oldest old surpassed 3.6 million, or about 2.8% of the total population in 2009, a 322% increase since 1990 [3]. Because the oldest old are vulnerable to age-related multiple chronic conditions and disabilities, and are at a high risk of losing their independence, considerable concern has arisen about the health care spending consumed by this age group. In a cross-sectional picture, the percentage of beneficiaries of long-term care insurance increased with age: for women, 2.2% aged 65-69, 5.4% aged 70-74, 12.8% aged 75-79, 27.1% aged 80-84, and 46.1% aged 85 and over; whereas for men it was 2.2% aged 65-69, 4.6% aged 70-74, 8.9% aged 75-79, 16.6% aged 80-84, and more than 30% aged 85 and over [4]. However, contrasting evidence suggests that living to an advanced age does not necessarily bring with it a high-cost dependency, but rather, that age-associated disability could be reduced at even the most advanced age by improvements in living conditions and health care delivery in certain countries [5,6]. These findings warrant testing in countries with a much higher pace of population aging.

The oldest population is characterized by its marked heterogeneity. Minimizing disability and dependency in the vulnerable part of this population is an undeniable public health priority. In contrast, engaging in social activities and having responsibility or roles in their communities is increasingly encouraged for their healthier counterparts in countries like Japan, where the fourth age (oldest old) is becoming a commonplace. Therefore, factors involved in retaining opportunities for community participation should be included in the scope of the forthcoming research. From this point of view, we have organized an interdisciplinary research team to investigate the multidimensional structure of health and its life-course trajectory, essential prerequisites for effective health and social care planning.

Despite a growing body of literature on geriatric research, data of the oldest old are complicated and some important domains have been neglected from an epidemiological perspective. First, although cardiovascular disease (CVD) is a primary cause of death and has a critical role in the development of disability, the predictability and management of CVD risk factors in this age group remains a matter of debate. Evidence from a clinical trial treating the very elderly with hypertension [7] and population-based observations [8,9] claim to develop an effective risk-stratification system. Incorporation of non-invasive measurement, such as carotid intimal medial thickness (IMT) will provide essential information about the pathological basis of atherosclerosis and help refine the risk assessment in this unique population. Second, nutritional status is critically important to the maintenance of health and function, yet little attention has been given to oral health, which surely affects daily energy intake and nutritional habits. Evidence suggests that poor oral health was associated with an increased risk for mortality [10,11], disability [12], dementia [13], CVD [14], and a variety of chronic diseases including diabetes, emphysema, hepatitis, osteoarthritis [15], and adverse drug effects [16]. Given the multipathologies among the oldest old, involvement of the dental profession in comprehensive geriatric research enables us to not only investigate the mechanisms underlying complex association between oral health and chronic conditions but also to help develop oral function-based nutritional intervention. Third, according to both increasing costs and older adults' preferences, long-term care policies are shifting away from institutional-based care system toward more home- and community-based services [17]. However, personal, social, and environmental resources that facilitate independence in home and community settings in the face of declining health and functioning (known as "aging in place") are not fully understood. Identifying environmental effects of factors such as social networks, community participation, and use of assistive technology on health and well-being, as well as clarifying the sustainability of health-related behaviours, plays an important role in the planning of community-based interventions for the oldest inhabitants. Our knowledge of the multidimensional structure of health and function will evolve to include the above-mentioned dimensions, each of which has assumed a central position in shaping preventive intervention and health promotion in this fastest-growing population.

### Aims

The Tokyo Oldest Old Survey on Total Health (TOOTH) is a longitudinal observational study designed to explore what happens to the physical, mental, and oral health of the community-living oldest old that incorporates non-invasive measurements of subclinical diseases, and to investigate factors that influence their health and health maintenance with a particular focus on the lifelong sustainability of healthy behaviours and social engagement.

The specific aims of the TOOTH are to:

1) Analyze the multidimensional structure of health and age-related functional decline with emphasis on an interdisciplinary team approach.

2) Quantify subclinical diseases including carotid atherosclerosis and osteoporosis by using non-invasive techniques and examine the impact of subclinical disease on functional decline and mortality.

3) Examine the association between oral health and functional decline, especially with regard to musculoskeletal function and nutritional status.

4) Assess the sustainability of health-related behaviours and social engagement and their impacts on maintenance of function and well-being.

## Methods

The TOOTH is a prospective observational study conducted by the interdisciplinary research scheme of Keio University, in which investigators from the Division of Geriatric Medicine, Department of Preventive Medicine and Public health, Faculty of Nursing and Medical Care, Faculty of Science and Technology, Faculty of Environment and Information Studies, and Faculty of Letters collaborated closely with Nihon University School of Dentistry. The TOOTH is designed to address key questions concerning the maintenance of health and function in community-dwelling seniors aged 85 years or older. Written informed consent was obtained either from the participants or by proxy (normally a family member or caregiver) when individuals lacked the capacity to consent. The study was approved by the ethical committee of the Keio University School of Medicine (N0.19-47, Dec 2007). TOOTH has been registered in the UMIN-Clinical Trial Registry (CTR) as UMIN-CTR (ID: UMIN000001842).

The TOOTH was designed using experience from the Tokyo Centenarians Study (TCS, individuals over 100 years of age) [18-20], with whom we share a common set of assessment and analytic procedures. The results from the TOOTH in combination with the TCS might help develop new models of preventive intervention and health promotion, which is likely to contribute to lifelong health and well-being.

### Sampling and recruitment

The base study population was comprised of male and female inhabitants of the wards of Shinjuku and Minato, and the east half of the Shibuya ward in the Tokyo Metropolitan area. The area of eligibility, which covers 6-km radius around the rehabilitation center at Keio University Hospital, was selected to facilitate door-to-door recruitment and limit transportation burden and cost. In January 2008, the area had approximately 14000 inhabitants aged 85 years or older (2.4% of the total population), which was comparable with the average of the Tokyo metropolitan area (2.3%).

Recruiting the oldest individuals to an epidemiological study that included an extended clinic examination was an unprecedented challenge. Therefore, we developed the following strategies to minimize the burden on participants and to enhance recruitment. First, evidence suggested that door-to-door recruitment was the most successful, particularly for the older population [21,22], so we employed this procedure for the TOOTH recruitment. Eligible individuals who were randomly selected from residences in the target area were sent an introduction letter informing them that they would be visited by an interviewer from the TOOTH project in one week to discuss participation in the study. Whenever a home visit did not result in contact with the potential participant or another member of the household, the interviewer left a note indicating how the study staff could arrange contact. Up to three attempts to establish the contact were made by an interviewer. If this was unsuccessful, no further attempts were made due to limited work force and resources. Once potential participants agreed to take part, written informed consent was obtained and subsequently an in-home and face-to face interview was conducted. Individuals who completed the interview session and expressed interest in the clinic examination were referred to the TOOTH study staff, who made telephonic arrangements for either a clinic visit or a home-based examination. Second, transporting the oldest individuals to our study center posed a commutational challenge not only due to mobility impairments but also participants' reluctance to visit huge and complicated facilities such as university hospitals. We addressed these barriers by establishing a door-to-door coach system. To secure the whole process of transportation to and from the study center and to alleviate participants' fear, a hired coach featuring a geriatrician provided door-to door transportation upon requested. A maximum of 8 individuals (except accompanying person) were transported by this coach system at any given time to limit the burden on board. Travel expenses were reimbursed to participants who visited the study center by the public transportation or taxi. Third, despite our maximum effort, a significant number of participants, possibly the more vulnerable subgroup, were unwilling to visit the study center. To capture a wide range of health within this age group, we provided the option of a home-based examination, in which participants were visited by a study team consisting of a geriatrician and a dentist. The home-based examination was functionally equal to the clinic examination with the exceptions of carotid atherosclerosis, bone mineral density measurements, and masticatory assessment using colour-changeable chewing gum. Finally, clinic- and home-based examinations were conducted in the spring (March to June) or in autumn (September to November) but not in the summer or winter considering the subjects' potential vulnerability to the weather.

### Baseline data collection

The baseline assessment consisted of an in-home interview, a self-administered questionnaire, and a clinic-based examination. In order to permit valid comparisons with other studies including the TCS and national and international epidemiological studies of the oldest old populations, the TOOTH deliberately adopted standardized and validated instruments of data collection. These instruments were pre-tested on a convenience sample of 10 volunteers in 2007 and were slightly simplified before the recruitment began. For the interview survey, participants were visited at their residences by field interviewers who had completed training in interviewing older persons. The interview session required on average of 47 minutes to complete depending on each subject's status including visual and hearing acuity and cognitive function. If participants felt fatigue during the interview, the session was stopped and a revisit was scheduled. After completion of the interview, the study staff contacted the individual by telephone to arrange either a clinic- or home-based examination and sent him or her a research information pack that included a letter describing the examination process, a photograph of the study staff, a detailed map of the study center at Keio University Hospital, self-administered questionnaires, and a special tube for urine collection. Participants were asked to complete the questionnaire and bring with them to the clinic visit. The clinic examination was conducted by the study team, which consisted of experienced geriatricians, dentists, and trained nurses and examiners at the rehabilitation center at Keio University Hospital. For those who were reluctant to visit the clinic, a home-based examination was scheduled. No pressure was put on the participants to choose either the clinic- or home-based examination.

### In-home interview

The baseline in-home interview was conducted by specially trained interviewers who collected extensive information on socioeconomic status, subjective health, behaviours, well-being assessed with the Philadelphia Geriatric Center (PGC) Morale Scale [23] and World Health Organization-5 (WHO-5) Well-being Index [24], cognitive function assessed with the Mini-Mental State Examination (MMSE) [25], basic activities of daily living (BADL) [26] and instrumental ADL (IADL) [27], a various measurements of social network and support, care needs, and usage of electrical home appliances (Table 1). These items were selected in order to capture the spectrum of health, functioning, and living conditions for the metropolitan Tokyo cohort aged 85 years or older.

**Table 1:** Contents of the baseline data collection

Components	Measurements
In-home interview	
Socioeconomic status	Marital status, composition of household, achieved education, current and past working activity, self-assessment of economic status
Functional capacity	Unintentional weight loss, vision and hearing, basic and instrumental activities of daily living
Behaviours	Smoking, alcohol use, walking
Well-being	Philadelphia Geriatric Center (PGC) Morale Scale
Mental health	World Health Organization (WHO)-5 Well-being Index
Cognitive function	Mini-mental state examination (MMSE)
Social cognition	Sense of control
Social intelligence/life knowledge	Wisdom
Social relations	Social networks, social support, negative interaction, companionship, reciprocity
Social activities	Content and number of social activities
Social care	Usage and degree of long-term care insurance
Usage of electrical home appliances	Microwave oven, washing machine, cell phone, personal computer, video game
	
Self-administered questionnaire	
Habitual dietary intake	46-item semiquantified food frequency questionnaire
Health-related QOL	WHO Quality of Life Instrument-Older Adults Module (WHOQOL-old)
Personality	NEO Five Factor Inventory (NEO-FFI)
	
Medical/dental Examination	
Chronic conditions	Medical history and medication
Falls	Falls in past 6 months
General pain	Visual analogue scale (VAS)
Health behaviors	Physical and cognitive activity, bowel habit
Physical examination	Resting blood pressure, heart rate, auscultation for the heart and the lung, detecting pitting ankle edema
Anthropometrical measurement	Weight, height, demi-span, waist, hip, calf circumference, skinfold thickness of triceps, subscapular, suprailiac, and medial calf
Physical function	Timed up-and-go test, chair standing, one-leg standing, grip strength
Electrocardiogram (ECG)	Twelve-lead ECG and 3-minute heart rate variability
Carotid atherosclerosis	B-mode carotid ultrasonography
Bone mineral density	Calcaneal quantitative ultrasonic measurement
Dental examination	Tooth count, degree of mouth cleaning, periodontal disease
Oral function	Bite force, masticatory function, 15-item food acceptance
Oral health-related QOL	General oral health assessment index (GOHAI)
Blood analysis	Non-fasting blood test and DNA sampling
Saliva analysis	Production and component of saliva
Urine analysis	Urine sample from the first morning boid (collected at home)

### Self-administered questionnaires

A set of self-administered questionnaires including a self-administered dietary history questionnaire [28], a health-related QOL (WHO Quality of Life Instruments-Older Adults Module, WHOQOL-old) [29], and a comprehensive personality inventory [30] was mailed to subjects who completed the interview session and were willing to take part in the clinic- or home-based examination. Eligible individuals were allowed to access the study staff by telephone when they found difficulties or obscurity in answering the questionnaires. Quality of data, coherence, and completeness were checked by an experienced geriatrician during the clinic- or home-based examination.

### The medical/dental examination

The medical/dental examination conducted at the rehabilitation center at Keio University Hospital included medical history, general pain, bowel habits, health behaviours, physical examination, anthropometric measurements, physical performance, 12-lead electrocardiogram followed by a 3-minute recording of heart rate variability (HRV), carotid ultrasonography, bone mineral density measurement, equipped dental examination, and the drawing of a venous blood sampling (Table 1). The examination required approximately 2.5 hours per subject to complete. At the beginning of the examination, the subjects were seated comfortably on a sofa and a team leader (Y.A. or N.H.) explained the examination and how the biological specimens would be analyzed and stored for future examination, subsequently obtaining written informed consent for both the procedure and DNA banking.

### Assessment

#### a) Comorbidity and medication

Information regarding comorbidities and medications was obtained from personal interviews and medical examinations conducted by geriatricians. Participants were asked to bring their prescriptions and other medical reports to the clinic examination so the geriatricians could refer to them. Diseases evaluated in this study included cerebrovascular disease, heart disease, hypertension, pulmonary tuberculosis and other respiratory disease, renal insufficiency, type 2 diabetes mellitus, dyslipidemia, Parkinson's disease, collagen disease, arthritis, osteoporosis, fractures, cataracts, gastrointestinal disease, and malignancies. The list of comorbidities used in the TOOTH were common to those in the TCS and the disease classification was based on the International Classification of Diseases, 10^th ^Revision, which enables us to compare the morbidity profiles of the TOOTH and the TCS cohorts.

#### b) Anthropometric measurement

Height, weight, demi-span, arm, waist, hip, and medial calf circumferences, and triceps, subscapular, suprailiac, and medial calf skinfold thicknesses were measured by a single examiner at the study center, or by a geriatrician during the home-based examination to minimize inter-observer variation.

#### c) Physical performance

To assess lower extremity performance, the timed up-and-go test [31], timed chair standing, and one-leg standing test [32] were measured. These measures have been well validated in older adults [33]. The tests were performed by two examiners, primarily a physical therapist and an assistant to prevent falls or injuries. To standardize the testing conditions, all measurements were conducted on a smooth surface and all subjects were barefooted. In the home-based examination, the timed up-and-go test was not applicable due to limited space. A suitable chair found in each participant's home was used for the chair standing test.

Handgrip strength of the dominant hand was measured in duplicate using grip strength measured using a handheld dynamometer (Tanita 6103, Tanita cooperation, Tokyo, Japan).

#### d) Carotid ultrasonography

Two-dimensional ultrasound examination of the bilateral common carotid artery was performed with a 10 MHz linear transducer (Hitachi EUB-525, Hitachi medical, Tokyo, Japan) following a standardized protocol [34]. All examinations were performed by a single physician, who was blinded from the subjects' clinical information. Definitions of the carotid segments were adopted from the Cardiovascular Health study [34]. An anterolateral approach was used to obtain a longitudinal image of the common carotid artery (CCA) and the internal carotid artery (ICA), and IMT of the far wall at each site was measured online as the distance between the luminal-intimal interface and the medial-adventitial interface. A thorough search of the common carotid artery and the internal carotid arteries was also made to determine the presence of focal plaque and/or calcific deposits. Plaque was defined as a clearly identified area of focal increased thickness (1.2 mm) of the intima-media layer.

#### e) Twelve-lead ECG and 3-minute HRV recording

After 15 minutes of supine resting for carotid ultrasonography, the 12-lead ECG and subsequent 3-minute ECG were recorded for HRV analysis (ECG-1550, Nihon Koden Ltd, Tokyo, Japan). Exclusion criteria for HRV analysis were: 1) <10% of ectopic beats; 2) atrial fibrillation/flutter; and 3) artificially paced-beats and other severe arrhythmia. In home-based examinations, the 12-lead ECG and 3-minute recordings were obtained using a portable ECG recorder (Cardiofax S, Nihon-Koden Ltd, Tokyo, Japan).

#### f) Measurement of bone mineral density

Calcaneal osteo-sono index (OSI) of the oldest-old using calcaneal quantitative ultrasonic measurements was used as a marker of bone mineral density. OSI is closely correlated with bone mineral density determined by dual X-ray absorptiometry (r = 0.87). An Achilles bone densitometer (AOS-100, ALOKA Co. Ltd., Tokyo, Japan) measured the speed of sound (SOS) and the transmission index (TI) of the right heel. OSI was calculated as follows:

The intra-observer coefficient of variation for OSI was 2.2% [35].

#### g) Dental examination

The dental examination was conducted by trained dentists from the Department of Complete Denture Prosthodontics, Nihon University School of Dentistry. The dental interview included a questionnaire about oral health care, oral health-related QOL evaluated by Geriatric Oral Health Assessment Index (GOHAI)[36], and a 15-item food acceptance section with three possible answers; with no difficulty, with caution, or no acceptance. The dental examination included the degree of mouth cleaning and periodontal disease (referred Debris Index and Calculus Index for judgment), condition of the temporomandibular joint (using the Japan Association of School Dentists index), number and condition of remaining teeth, and the condition of any dental prostheses. Oral function was assessed by two measurements; bite force and masticatory ability using colour-changeable chewing gum (Kracie Foods, Ltd., Tokyo, Japan). The bite force of maximal voluntary clenching was measured on the dominant side using a bite force measuring device (Occlusal Force-Meter GM10, Nagano Keiki Inc., Tokyo, Japan). The colour-changeable chewing gum test was non-invasive and demonstrated to be consistently associated with masticatory ability in the elderly [37-39]. In this study, we measured the change of the colour (from green to red) after 90 chews using a spectrophotometer (CM-2600d, Konica Minolta Inc., Osaka, Japan). Although the risk of aspiration posed by this procedure was estimated to be very limited, we did not use this measurement in participants who had swallowing dysfunction or in any of the home-based examinations.

#### h) Laboratory analysis

Participants were asked to collect urine samples from the first morning void of the examination day into a special tube. Non-fasting venous blood was sampled to collect serum, EDTA plasma, citrate plasma, EDTA full blood, and a subsample for RNA extraction. Biological specimens including urine, blood, and saliva were stored at -80° until subsequent analysis.

Home-based examinations, in which participants were simultaneously visited by a geriatrician and a dentist, were conducted the same way as the clinic-based examinations with the exception of carotid ultrasonography, and bone mineral density measurements.

### Follow-up

Participants will be followed-up by an annual telephone interview by the study staff. The annual follow-up purports to study outcomes such as subjective health, institutionalization, morbidity, sustainability of health behaviours and social participation, disability in instrumental and basic ADL, and death. In addition, participants are submitted every second year to an in-home interview to study objective changes in physical and mental function. A planned follow-up study will allow further clarification of the determinants of mortality and functional decline among the oldest old.

### Data analysis

All questionnaire, interview, and examination forms were doubly checked by trained researchers. Data entry quality has been systemically monitored to detect errors. All statistical analysis was performed by SPSS 16.0 software package (SPSS, Chicago, IL). Baseline demographics and health measures were presented using descriptive statistics. Statistical analysis included: cross-tubular comparison using unpaired t-tests, analysis of variance (ANOVA), or Kruskal-Wallis analysis, calculations of the correlation coefficients, and linear and logistic regression analysis; prospective analyses for the risk factors for functional decline and mortality are planned using Cox proportional hazards methods. The variety of dimensions included in the TOOTH study will enable us to control for a wide range of factors in multivariate models.

#### Sample size considerations

To analyze the multidimensional components of health and functioning, the TOOTH study examines a large number of specific measurements. Therefore, formal sample size calculations for the whole study were not feasible. A sample size of 542 for the in-home interview plus the medical/dental examination (if divided into two groups by a factor, 271 each) for the in-home interview plus the medical/dental examination provides a sufficient power of 0.8174 to detect as significant with alpha of 0.05 (two-sided), 30% reduction in mortality (40% vs. 27%) in the 5-year follow-up period based on the previous studies [40,41]. The power increases to 0.9891 when we use full sample (n = 1152).

## Results

### Recruitment

**Figure 1:**
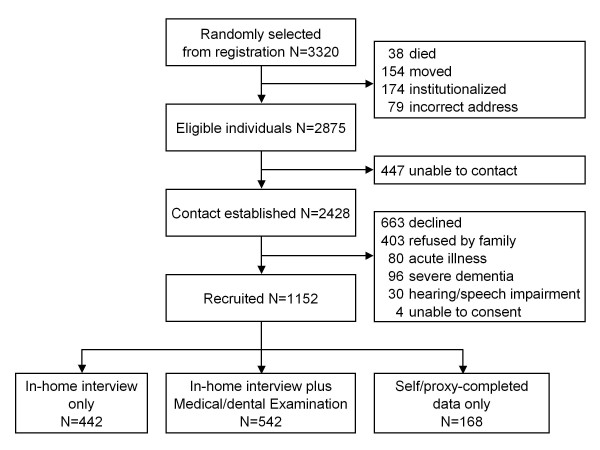
**Recruitment flowchart of the TOOTH cohort**.

The TOOTH sampling and recruitment is summarized in Figure 1.

A total of 3320 individuals born before January 1923 (1600 in January 2008, 1720 in January 2009) were randomly drawn from the basic registry of residents of each ward. Recruitment began in March 2008; by the end of November 2009, a total of 1152 people were recruited, of which 542 completed both the in-home interview and the medical/dental examination, and 442 completed the in-home interview only. Another 168 subjects, who were unwilling to give a face-to face interview, agreed to provide self- or proxy-completed data on sociodemographics, basic and instrumental ADLs, and usage of social care. Among those who completed the medical/dental examination, 460 (84.9%) individuals visited our study center, and 82 (15.1%) participated in the home-based examination. Participants of the home-based examination chose not to visit the study center due to walking disability (n = 52), reluctance to leave their home (n = 17), severe illness (n = 5), being the prime caregiver for their spouse or grandchildren (n = 3), or severe dementia (n = 3).

### Baseline characteristics

Baseline characteristics of the 542 participants who completed both the interview and the examination, the 442 who participated in the interview session only, and the 168 who provided only self- or proxy-completed data are shown in Table 2. Compared to those who completed the interview session only, older (90+) and female individuals were underrepresented in the examination cohort. Examination participants were more likely to be better educated, have a better MMSE score, and be less disabled. In contrast, self-rated health was comparable between the two groups. Compared to the interview group and the interview plus examination group, those who provided self- or proxy-completed data only were obviously characterized by a higher percentage of 90+, of fair-to-poor rating in subjective economic status, and of disability in BADL and IADL.

**Table 2:** Background characteristics of the study participants according to the recruitment scheme

		In-home interview only		In-home interview plus Medical/dental examination		Self/proxy completed data only	
		n = 442		n = 542		n = 168	
Gender	Male	26.0	(115)	43.5	(236)	26.2	(44)
	Female	74.0	(327)	56.5	(306)	73.8	(124)
Age	85-89	78.5	(347)	91.5	(496)	48.2	(81)
	90-94	18.1	(80)	7.0	(38)	41.1	(69)
	95-99	2.9	(13)	1.1	(6)	9.5	(16)
	100+	0.5	(2)	0.4	(2)	1.2	(2)
Living condition							
alone		40.6	(179)	34.0	(183)	NA	
with spouse only		28.6	(126)	27.0	(145)	NA	
with others		30.7	(135)	39.0	(210)	NA	
Education							
Higher education		10.6	(47)	20.1	(109)	NA	
Self-rated health							
Excellent to good		52.2	(230)	51.0	(274)	NA	
Fair to poor		47.8	(211)	49.0	(263)	NA	
Subjective economic status							
Excellent to good		62.2	(255)	72.8	(378)	57.4	(93)
Fair to poor		37.8	(155)	27.2	(141)	42.6	(69)
MMSE							
mean (SD)		23.7	(4.9)	26.2	(3.9)	NA	
median [IQR]		24	[21-27.5]	27	[24-29]	NA	
Barthel index							
mean (SD)		78.7	(31.4)	97.1	(8.2)	56	(37)
median [IQR]		95	[70-100]	100	[95-100]	60	[20-95]

Notes: Values are expressed as percentage (numbers) unless otherwise stated.

NA; not applicable, SD: standard deviation, IQR; interqurtile range, MMSE; Mini-mental state examination.

### Feasibility of non-invasive measurements of subclinical diseases

Carotid ultrasonography was completed in 458 subjects, which was 84.5% of the examination participants (n = 542) and 99.6% of the clinic visitor (n = 460). The reason for non-applicability of the procedure was kyphosis (n = 2). Collection of bone mineral density measurements became available in April 2009, and 275 subjects (99.3% of eligible subjects) were tested.

Regarding the oral health examination, assessment of masticatory ability using a colour-changeable chewing gum was conducted in the clinic setting only, and 421 subjects (91.5% of the clinic visitors) completed this procedure. The reasons for non-applicability were existing dental diseases (n = 12), unfitness of dentures (n = 10), oral pain (n = 3), inability to participate (n = 5), misunderstanding of the procedure (n = 2), and refusal (n = 5).

No participants reported an adverse event as a result of participation in any assessment in either the clinic- or home-based examination.

## Discussion

The TOOTH study has a stated aim to create a database to allow for detailed analysis of physical, mental, and oral health and modifiable determinants of maintaining health, independence, and community participation. It includes a range of key questions not addressed in previous studies, particularly with regard to a detailed analysis of oral function and non-invasive assessment of subclinical diseases. Inclusion of a quantifiable measure of atherosclerosis will provide mechanistic insights into the association between progression of disease process and classical and novel risk factors, which enables us to refine the risk stratification and to investigate the effects of subclinical atherosclerosis on age-related functional deterioration and mortality. In addition, longitudinal observation of multidimensional measures of health, participation and behaviours will clarify the impact of social participation as a worker, volunteer, or community member on maintenance of health and well-being, hence offering new avenues for community interventions in later life.

In this paper, we demonstrated feasibility of a new comprehensive study that incorporated non-invasive measurements of subclinical diseases and detailed dental examination focusing on individuals aged 85 years or older living in the community. The methodological design, which enabled us to standardize assessments of physical performance and anthropometric measurement at the clinic site, was also strength of the TOOTH study. In recruiting the oldest individuals to our study center, we made all possible efforts to minimize all obstacles both real and perceived to coming to the study centers, particularly in securing their transportation. In addition, in-home examination by a geriatrician and a dentist was conducted for those who were reluctant to visit our study center, which enabled us to incorporate a relatively frail segment of this population. Therefore, a bimodal recruitment strategy is critically important not only to increase the participation rate but also to capture a broad range of health profiles among the oldest old.

There were several limitations in our study. First, a significant number of female and older (90+) individuals were underrepresented in the examination cohort; resulting raised proportion of male and relatively well-functioning counterparts in the population. Longitudinal observation of this cohort will allow us to capture the pathways from health to disability; however, this may limit the generalizability of our results. Second, in the recruitment procedure, we were unable to make contact with a significant number of individuals. Previous evidence reported that using telephone for the establishment of the initial contact could effectively reduce non-contact rates [42]; however, we strictly inhibit this strategy due to ethical reason. Telephone-based swindling that randomly targets elder individuals has been burgeoning in the metropolitan area, and unfamiliar telephone calls are becoming detestable and stressful to the senior inhabitants, and even turbulent for those with cognitive impairment. Therefore, we relied exclusively on the face-to-face recruitment by trained interviewers. Third, some of the non-invasive measurements were not applicable in home-based examinations, which could lead to an underestimation in the association between subclinical diseases and disability. Future improvement in measurement devices will address one aspect of this issue; however, limitation of human (examiner) resource remains.

Since the pioneering work from the Leiden 85-Plus Study [43], increasing research efforts have been aimed at the oldest old [44-47]. However, substantial uncertainty and complexity remain to be elucidated. The results of the TOOTH has the potentials to yield a wealth of important information in previously underexposed components of health and well-being, and to enhance the effective health care delivery and community participation in the fourth stage of life.

## Competing interests

The authors declare that they have no competing interests.

## Authors' contributions

YA directed the conceptualization and design, execution of the study, data collection, analysis, and interpretation, and drafted this paper. TI contributed to the design, data collection, and directed the dental examination and writing of the paper. MT contributed to the design, execution of the study, data collection, analysis, and writing of the paper. MT contributed to the design, directed data collection of social network and community participation, and writing of the paper. YA contributed to collection of biological specimen, and directed the data management. RF, JA, KO, and HH contributed to the design, development of questionnaires. KA and YN contributed to the design, recruitment strategy, data analysis, and writing the paper. YG and HA contributed to the design and establishing assessment inventory for social network and participation. KK and NG provided oversight to all aspects of the dental examination, data collection, and analysis. NH oversaw all the aspects of the TOOTH study, and contributed design, data collection. All authors read and approved the final manuscript.

## Pre-publication history

The pre-publication history for this paper can be accessed here:

http://www.biomedcentral.com/1471-2318/10/35/prepub
